# Differentiating comorbidities and predicting prognosis in idiopathic normal pressure hydrocephalus using cerebrospinal fluid biomarkers

**DOI:** 10.3325/cmj.2021.62.387

**Published:** 2021-08

**Authors:** Madoka Nakajima, Kaito Kawamura, Chihiro Akiba, Koichiro Sakamoto, Hambing Xu, Chihiro Kamohara, Ikuko Ogino, Kostadin Karagiozov, Yuichi Tange, Kazuaki Shimoji, Shinya Yamada, Akihide Kondo, Hajime Arai, Masakazu Miyajima

**Affiliations:** 1 Department of Neurosurgery, Juntendo University Faculty of Medicine, Tokyo, Japan; 2 Department of Neurosurgery, Juntendo Tokyo Koto Geriatric Medical Center, Tokyo, Japan; 3 Department of Neurosurgery, Kugayama Hospital, Tokyo, Japan

## Abstract

Idiopathic normal pressure hydrocephalus (iNPH) is a condition resulting from impaired cerebrospinal fluid (CSF) absorption and excretion characterized by a triad of symptoms comprising dementia, gait disturbance (impaired trunk balance), and urinary incontinence. CSF biomarkers not only assist in diagnosis but are also important for analyzing the pathology and understanding appropriate treatment indications. As the neuropathological findings characteristic of iNPH have yet to be defined, there remains no method to diagnose iNPH with 100% sensitivity and specificity. Neurotoxic proteins are assumed to be involved in the neurological symptoms of iNPH, particularly the appearance of cognitive impairment. The symptoms of iNPH can be reversed by improving CSF turnover through shunting. However, early diagnosis is essential as once neurodegeneration has progressed, pathological changes become irreversible and symptom improvement is minimal, even after shunting. Combining a variety of diagnostic methods may lead to a more definitive diagnosis and accurate prediction of the prognosis following shunt treatment. Identifying comorbidities in iNPH using CSF biomarkers does not contraindicate shunting-based intervention, but does limit the improvement in symptoms it yields, and provides vital information for predicting post-treatment prognosis.

## Background

Idiopathic normal pressure hydrocephalus (iNPH) is a syndrome that occurs in older adults without the incidence of meningitis, subarachnoid hemorrhage, or other antecedent conditions. This syndrome is caused by impaired cerebrospinal fluid (CSF) absorption, primarily leading to gait disturbance, cognitive impairment, and urinary incontinence (1). It is a condition with great variation in presentation and no standard clinical configuration of symptoms. The most well evidenced treatment method is CSF shunting, yet its results are not consistent, even within the same disease group, due primarily to comorbid neurodegenerative disease.

The goal of measuring CSF biomarkers is to facilitate the accurate diagnosis of iNPH as an auxiliary diagnostic tool and predict shunt treatment prognosis. In this review, we discuss potential CSF biomarkers that may be used to differentially diagnose iNPH and predict prognosis following shunt treatment.

## Classification of normal pressure hydrocephalus

**Figure 1:**
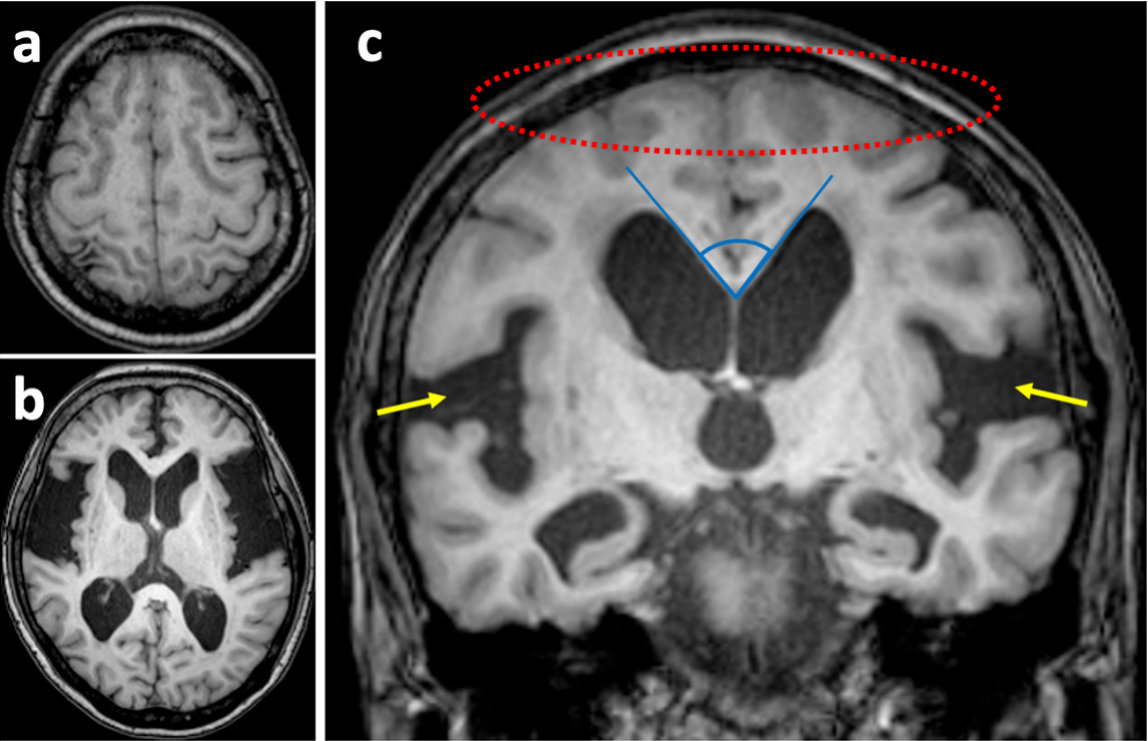
Representative magnetic resonance imaging (MRI) of disproportionately enlarged subarachnoid space hydrocephalus (DESH). Ventriculomegaly, (**A**) tight sulci in the midline and the high convexity and (**B**) dilated Sylvian fissures are demonstrated. (**C**) The callosal angle is the angle between the lateral ventricles measured on the coronal plane, which is perpendicular to the anterior-posterior commissure plane through the posterior commissure of the patients.

Patients aged 60 years or more exhibiting any one symptom of the symptom triad (gait disturbance, cognitive impairment, or urinary incontinence) and showing cerebral ventricular enlargement (Evans index >0.3) on head computed tomography or magnetic resonance imaging (MRI) are classified as possible iNPH (2). Patients with typical gait disturbances (wide-base gait, small-step gait, or “magnetic” gait) as a result of poor balance and presenting disproportionately enlarged subarachnoid space hydrocephalus (DESH) (3,4) are classified as probable iNPH with MRI support according to Japanese iNPH guidelines (2) (Figure 1). If radiological signs (iNPH Radscale) reflect the presence of DESH findings, that could accurately differentiate between patients with definite iNPH and asymptomatic individuals above the age of 65 (5,6).

Japanese guidelines (2) differentiate iNPH from the various types of late-onset congenital hydrocephalus, such as panventriculomegaly with a wide foramen of Magendie and a large cisterna magna (PaVM) (7), longstanding overt ventriculomegaly in adults (LOVA) (8), late-onset idiopathic aqueductal stenosis (LIAS) (9-11), and late-onset aqueductal membranous occlusion (LAMO) (12) demonstrating advanced ventricular enlargement. The international iNPH guidelines do not require DESH findings for diagnosis but instead incorporate these disease groups within iNPH and define a much younger age of 40 years or older for initial onset (13).

LOVA presents with an enlargement of the lateral and third ventricles due to congenital cerebral aqueduct obstruction and consequences of a long-term increase of intracranial pressure, including head circumference increase and sella turcica destruction, as well as cognitive impairment, abnormal gait, and urinary incontinence in adulthood (8). As LOVA is a non-communicating hydrocephalus due to cerebral aqueduct stenosis, the effective treatment is either with endoscopic third ventriculostomy (ETV) (14), or shunt surgery if the patient has a reduced ability to absorb CSF. On the other hand, PaVM is a communicating hydrocephalus resulting from congenital occlusion or impaired circulation in the foramen magnum area. In addition to Blake’s pouch cyst (15,16) or extraventricular intracisternal obstructive hydrocephalus (17), PaVM features atresia of the foramen of Magendie and has a range of presentations from asymptomatic to symptoms resembling the NPH triad. In some recent international studies on the effectiveness of ETV for iNPH (18), it is likely that the patients who received treatment included multiple disease groups, and the results can only be discussed considering the differences in the definition of iNPH between Japan and other countries.

**Figure 2:**
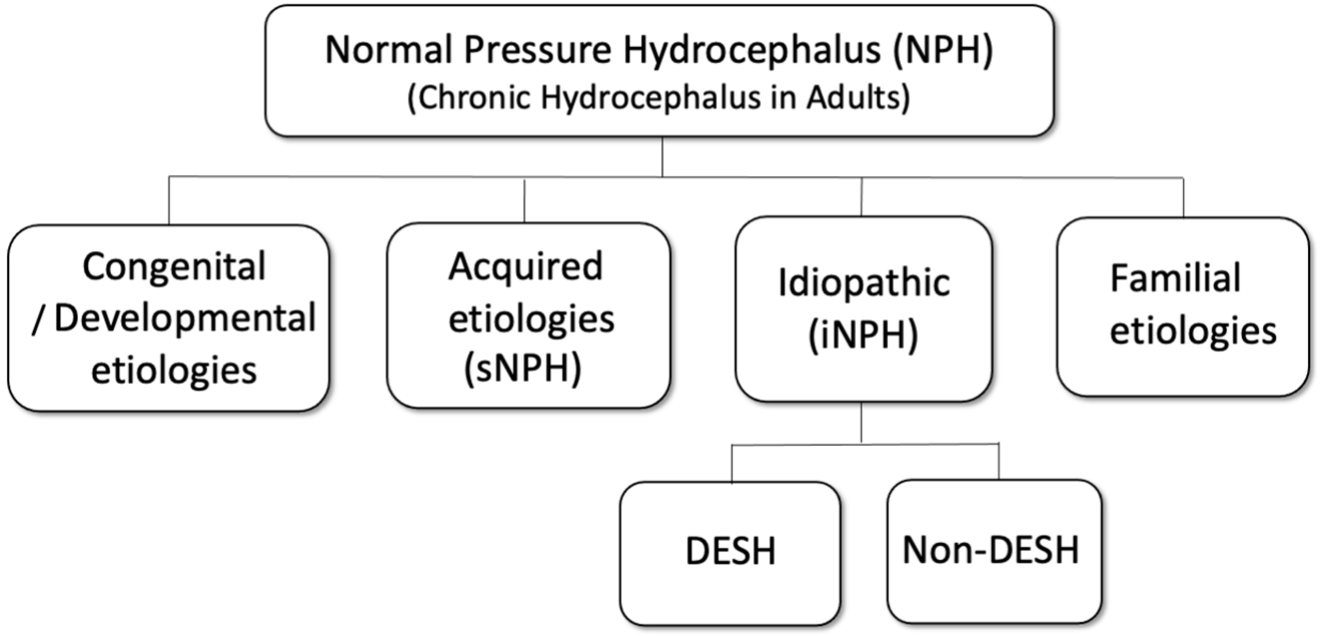
Classification of chronic hydrocephalus in adults. DESH – disproportionately enlarged subarachnoid space hydrocephalus; sNPH – secondary normal pressure hydrocephalus.

In this review, we differentiated between iNPH groups and NPH accompanied by congenital or developmental abnormalities (Figure 2). In addition to diagnostic imaging, a study found that CSF biomarkers assist differential diagnosis. CSF protein tyrosine phosphatase receptor type Q (PTPRQ) concentrations were significantly higher in NPH with congenital/developmental etiologies compared with the iNPH group (19). PTPRQ is expressed in ependymal cells and the choroid plexus. It is highly possible that the higher PTPRQ levels in the CSF are related to ependymal dysfunction from ventricular expansion.

## Diagnosis of idiopathic normal pressure hydrocephalus

The Japanese iNPH guidelines (2) focus on “tight high convexity and medial sulci/subarachnoid spaces” as the major feature of iNPH. A Japanese prospective cohort study, known as the Study of Idiopathic Normal Pressure Hydrocephalus on Neurological Improvement (SINPHONI) (4), found a response rate of 80% based on changes to the modified Rankin Scale score, and therefore determined that shunting was best performed without a prior tap test if there are findings suggestive of DESH and gait disturbance (20).

**Figure 3:**
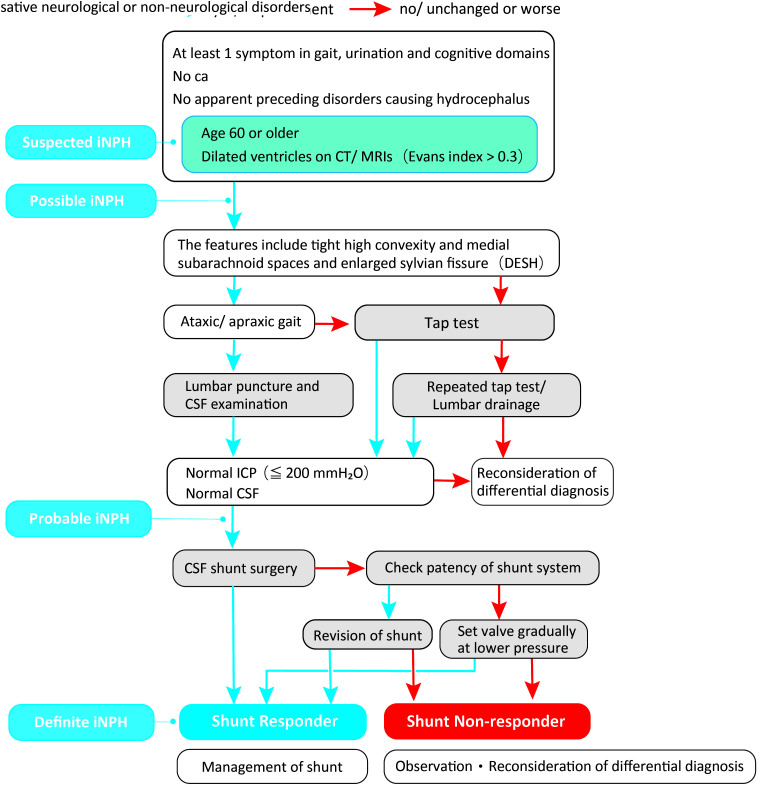
Flowchart for idiopathic normal pressure hydrocephalus (iNPH) diagnosis. CT – computed tomograpy; MRI – magnetic resonance imaging; DESH – disproportionately enlarged subarachnoid space hydrocephalus; CSF – cerebrospinal fluid; ICP – intracranial pressure.

Even so, when diagnosed based on typical gait disturbance and imaging findings (possible iNPH with MRI support), it is suitable to perform a tap test along with CSF testing. In actual clinical settings, tap tests are performed in most cases in which iNPH is suspected. A tap test refers to a procedure in which approximately 30 mL of CSF is extracted through a lumbar puncture (using a 19-gauge or larger needle) to observe whether there is an improvement in symptoms, such as gait disturbance. Patients who show such an improvement due to CSF removal (tap test-positive) can be expected to obtain therapeutic effects from shunting (21). A tap test is essential in cases where there is doubt about the diagnosis, for example, when the patient has particularly atypical symptoms or a comorbid condition is suspected. Nonetheless, tap test sensitivity is approximately 60% (22), and it is acceptable to perform shunting even with a negative tap test result if the patient can be diagnosed with possible iNPH with MRI support based on symptoms and imaging findings (Figure 3). However, the post-shunting prognosis is inferior to that of patients with a positive tap test result. Another option is to determine prognosis based on the improvement in symptoms following a second tap test or a continuous lumbar drainage test (drainage of 100-300 mL CSF per day for 3-5 days) (22).

## Diagnosis with CSF biomarkers

### General remarks

iNPH in older adults must be carefully differentiated from other conditions that can cause cognitive impairment, gait disturbance, or both, and pathologies resulting in ventricular enlargement on imaging, that is, conditions exhibiting cerebral atrophy (23).

Specific conditions that must be ruled out are Alzheimer’s disease (AD), subcortical ischemic vascular disease (SIVD), and parkinsonian syndromes (Parkinson’s disease [PD], Lewy body dementia [LBD], frontotemporal dementia [FTD], progressive supranuclear palsy [PSP], corticobasal degeneration [CBD], multiple system atrophy [MSA]). It is difficult to clearly differentiate between iNPH and other diseases and it should be assumed that both iNPH and comorbid pathology can be present. Differences in clinical symptoms, such as the nature of the gait disturbance or cognitive impairment, and imaging findings are essential to differential diagnosis, but there are many cases in which decision making is difficult. Thus, in such cases using biomarkers to assist with diagnosis is considered particularly useful.

Biomarkers are substances, such as proteins, the concentrations of which reflect the existence and progression of a disease. Enzyme-linked immunosorbent assay (ELISA) is a method in which a target antigen or antibody is detected and quantified by applying a specific antibody or antigen and observing the enzymatic reaction. Ideal biomarkers for neuropathological diseases are those that: 1) can detect the essential characteristics of specific neuropathology and 2) are validated in patients with a definitive neuropathological diagnosis. Additional ideal conditions include 3) sensitivity >80%, 4) high reliability and reproducibility, and 5) non-invasiveness, low price, and simplicity (24).

Because typical neuropathological characteristics of iNPH have yet to be established, no biomarkers satisfy all the above conditions. However, many substances have been examined as potential CSF markers using CSF obtained during diagnostic pressure and quality examinations for the purpose of 1) diagnosis/differential diagnosis of iNPH and 2) prediction of the effects of shunting.

Many previous studies do not clearly differentiate between the cases of iNPH and secondary normal pressure hydrocephalus (sNPH), though studies focusing strictly on iNPH and investigations of CSF proteins, neuropeptides, microRNAs (miRNAs), and other potential markers are under way. Unfortunately, most of these studies have had small sample sizes, and few have been re-examined for reproducibility; as such, the evidence level remains low. As there is currently no method of diagnosing iNPH with 100% sensitivity and specificity, it is possible to make a more definitive diagnosis and predict the post-shunting prognosis by combining multiple diagnostic methods.

### Detailed investigation

The goal of diagnosing iNPH using CSF biomarkers also includes differentiation from healthy older adults and neurodegenerative disorders that may influence the prognosis of treatment interventions. Table 1 summarizes the current biomarkers that can differentiate between iNPH, AD, and healthy individuals, as well as predict the effectiveness of shunting.

**Table 1:** Cerebrospinal fluid (CSF) biomarkers for differentiating idiopathic normal pressure hydrocephalus (iNPH) from healthy individuals and Alzheimer’s disease, and for predicting CSF shunt effect*

Biomarker	Differential diagnosis of iNPH	CSF shunt effect prediction
Aβ42	No change compared to Alzheimer’s disease, lower than healthy individuals	Poor prognosis with decrease
HPt	Lower than Alzheimer’s disease, no change compared with healthy individuals	Poor prognosis with elevation
Total tau	Lower than Alzheimer’s disease, no change compared to healthy individuals	Poor prognosis with elevation
NfL	Higher than healthy individuals	Poor prognosis with elevation
Aβ38	No change compared to Alzheimer’s disease, lower than healthy individuals	
Aβ40	No change compared to Alzheimer’s disease, lower than healthy individuals	
LRG	Higher than healthy individuals	
PTPRQ	Higher than healthy individuals	
CSF-derived Tf-1	Lower than healthy individuals	

*Abbreviations: Aβ – β-amyloid; HPt – hyperphosphorylated tau; LRG – leucine-rich α2-glycoprotein; NfL – the light subunit of neurofilament triplet protein; PTPRQ – protein tyrosine phosphatase receptor type Q; Tf – transferrin; CSF – cerebrospinal fluid; iNPH – idiopathic normal pressure hydrocephalus.

#### Differentiation from healthy older adults

Being aware of the differences between the CSF of healthy older adults and those with iNPH is key to understanding the neuropathology of iNPH. The CSF in iNPH contains significantly increased levels of transforming growth factor-β signal transmission 1 (TGF-β1) and TGF-βII receptors (TβR-II) (25,26), which encourage angiogenesis; the light subunit of neurofilament triplet protein (NfL) (27-29), which is specifically distributed within neurons; and the known angiogenic glycoprotein, leucine-rich α2-glycoprotein (LRG) (30). Increased LRG has been confirmed in patients with definite iNPH from at least two facilities through comprehensive proteomic analysis (31-33). Additionally, levels of acetylcholine esterase activity, lactic acid (34), β-amyloid 1-42 (Aβ42) (35), Aβ38, Aβ40, lipocalin-type prostaglandin D synthase (L-PGDS) (36), secreted β-amyloid precursor protein (APP), and secreted APPα are also significantly lower in patients with definite iNPH (37). Secreted APPα has been studied in patient groups in Europe and Japan (38-41). It is useful as a marker due to its high reproducibility, but its correlation with iNPH pathophysiology has not yet been clarified. L-PGDS, primarily produced in the arachnoid membrane and present in high concentrations in CSF, has wide ligand-binding sensitivity and functions as both a transport protein for hydrophobic small molecules and a scavenger. L-PGDS positively correlates with total tau protein and can be used to differentiate between DESH and non-DESH types of iNPH (42). Recently, a rapid method for measuring the diagnostic marker candidate transferrin was developed, and it has been shown to be capable of predicting the effectiveness of shunting at multiple facilities with multiple specimens (43,44) (Table 1).

#### Differentiation from Alzheimer’s disease

AD is a major form of neurodegenerative dementia, but there is currently no effective treatment method for halting its progression. One reason is that a definitive diagnosis is generally not reached until too late into the disease progression. The clinical diagnosis of AD is based on neurological signs (cognitive impairment) and neuropsychiatric profiling of biomarkers (45). Definitive histological diagnosis that can confirm neuronal hyperphosphorylated tau (p-tau) and neural network aggregation of Aβ can only be made after the pathology has progressed to the terminal level and is therefore only performed on autopsy.

Regarding cognitive impairment, it is important to differentiate iNPH from AD, which is also the most common comorbidity of iNPH. However, one study in which frontal lobe biopsy was conducted during shunting found that participants exhibited Alzheimer’s pathology, with deposition of Aβ (44%) and tau protein (9%), thereby suggesting a high rate of comorbidity with iNPH (46).

Patients often do not develop the symptoms of gait disturbance or urinary incontinence until the advanced stages of AD. Forgetfulness is a core symptom of cognitive disorders, while persecutory delusions (eg, delusions of theft) are common peripheral symptoms. While the level of forgetfulness in iNPH is comparatively low, recognition is often maintained, and instances of delirium are limited. However, when AD and iNPH are comorbid, diagnosis from symptoms alone is difficult, and the features of each pathology must be ruled out through diagnostic imaging, such as MRI. Deposition of amyloid protein in the brain begins before the onset of AD and is, in fact, thought to be the pathogenesis. Positron emission tomography amyloid imaging has confirmed that AD shows abnormal amyloid deposition and CSF markers, lower Aβ42, and elevated total tau protein and p-tau compared with healthy older adults and iNPH (47-51). Thus, these are currently the most widely used biomarkers. Differences between facilities are unlikely, as all of them use the same commercial ELISA assay (INNOTEST^TM^, Innogenetics, Gent, Belgium). Patients with poor cognitive function improvement after shunting had higher p-tau, higher Aβ38/Aβ42 ratio, and lower Aβ42/ p-tau ratio than patients showing good improvement (36), suggesting that comorbid Alzheimer’s pathology contributes to cognitive function improvement after shunting in iNPH.

Apolipoprotein E ϵ4 (APOE4), a known risk factor for AD, has been linked to a high level of amyloid plaques in biopsied cortical tissue. However, the APOE-related risk of AD in patients with iNPH is not higher than that in the general population (52) and APOE4 is also not a risk factor for post-shunting prognosis (53). While comorbid AD influences post-shunting prognosis, particularly cognitive function prognosis (36), little research offers strong evidence for determining the true extent of its effects or whether shunting is a meaningful treatment. A multicenter, prospective, randomized study on the effectiveness and safety of shunting for patients suspected to have Alzheimer’s pathology is under way in Japan (SINPHONI-3, UMIN000035377).

#### Differentiation from subcortical ischemic vascular disease

MRI and single photon emission tomography (regional cerebral blood flow) show chronic ischemia in iNPH (54). Subcortical ischemic vascular disease (SIVD) is an extremely common comorbidity of iNPH. Pathological findings of iNPH also show ischemic lesions and sclerotic changes to the vascular walls in the cerebral parenchyma. Precisely classifying both conditions from symptoms alone is even more difficult than the differential diagnosis of AD. Association with cerebrovascular disorder is a risk factor for a diminished effect of shunting (55), but it is not a contraindication.

A single-center study found that the concentration of glycolipid sulfatide was elevated in SIVD, making it useful for differentiating from iNPH. The level of vasoactive intestinal peptide, a neuropeptide, in the ventricular CSF has also been shown to be lower in iNPH (29,56). Neuron-derived NfL and Aβ42 are also reliable biomarkers for differentiating iNPH from SIVD (57). There are reports of other markers, such as the synaptic protein neurogranin and L-PDGS, but these have low accuracy as tools for differential diagnosis.

#### Differentiation from Parkinson’s spectrum disorders

*Parkinson’s disease*. For comparisons with PD, we focused on differences in gait symptoms. Impaired postural reflexes and falling can be seen in the early stages of iNPH, but they are rare in the early stages of PD. Furthermore, resting tremor, masked facies, and speech abnormalities are uncommon in iNPH. The typical diagnostic imaging of PD includes dopamine transporter (DAT)-SPECT with ^123^I-ioflupane detecting decreased accumulation in the striatum, and myocardial perfusion scintigraphy with the noradrenaline analogue ^123^I-metaiodobenzylguanidine (MIBG) also detecting decreased accumulation. However, by definition, iNPH shows no abnormalities on these tests. Nonetheless, as is the case with AD, comorbidity of PD and iNPH is not uncommon (58), and even the confirmation of DESH findings cannot rule out comorbid PD (59). If comorbidity is suspected, carefully determining the extent of iNPH involvement in the present symptoms using the response to a tap test and observing the reaction to dopamine replacement therapy for PD (L-dopa test) can aid diagnosis (60).

Regarding biomarkers, many studies have reported that tau protein and Aβ are insufficient for differentiation (61). An older study was able to distinguish NPH from PD using a diazepam-binding inhibitor, which is higher in NPH and lower in PD (62). More recently, a reduction in α-synuclein (63) and differences in glycans that attach to transferrin (Tf) have been found to be useful in differential diagnosis (64,65). One study used the ratio of serum-derived Tf-2 and CSF-derived Tf-1 secreted from the choroid plexus (Tf-2/Tf-1) to successfully differentiate PD and MSA, in which Tf-1 levels are lower, from iNPH and healthy individuals.

*Lewy body dementia*. Visual hallucinations are a characteristic symptom of LBD that is rarely encountered in cases of iNPH. Another feature of LBD is fluctuating cognition, which can occasionally be drastic, but such sudden and major fluctuations are uncommon in iNPH. Cerebral blood flow SPECT findings of reduced blood flow in the occipital lobe and reduced accumulation in MIBG myocardial scintigraphy indicate LBD comorbidity. If such indications are confirmed and there are also findings of DESH, clinicians will first determine the contribution of iNPH to the current symptoms using the reaction to a tap test, followed by observing the reaction to treatment with cholinesterase inhibitors. The same fundamental biomarkers are used for LBD and PD (63). A recent study found higher levels of LRG in PDD and PSP than in iNPH (31). miR-3675, miR-4274, and miR-4310 were found to be useful in distinguishing between these conditions. miR-4274 and miR-4310 are involved in dopamine receptor signaling. Receiver operatic characteristic analysis confirmed that miR-4274 can be used to differentiate iNPH complicated by parkinsonian diseases to a high degree of accuracy (area under curve = 0.913) (66).

**Figure 4:**
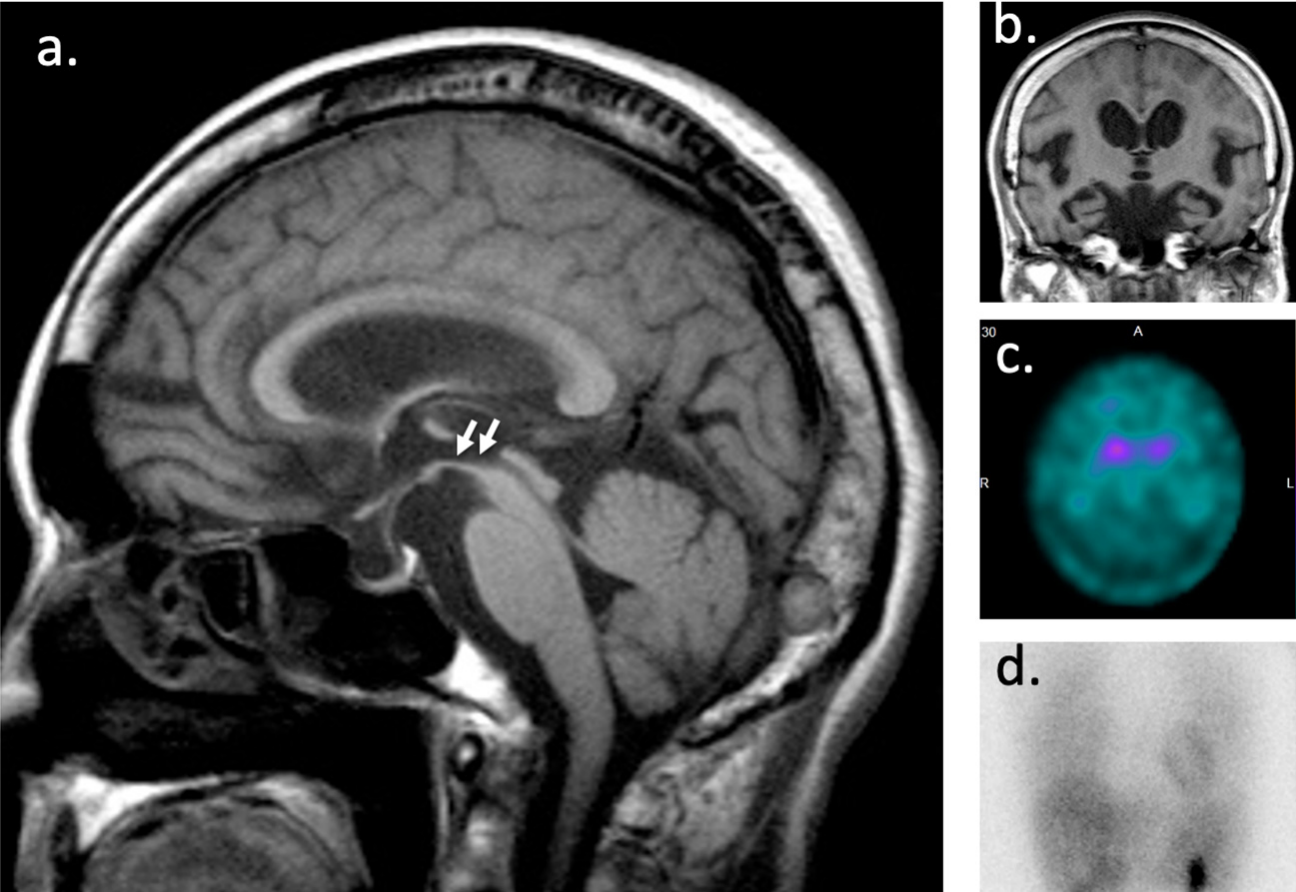
Progressive supranuclear palsy with disproportionately enlarged subarachnoid space hydrocephalus (DESH). (**A**) Sagittal section of brain magnetic resonance imaging (MRI) in a progressive supranuclear palsy (PSP) patient showing midbrain atrophy with preservation of the pons (below white arrows) known as the “hummingbird” sign. (**B**) Coronal section of brain MRI showing “DESH” sign. Dopamine transporter imaging with [^123^I] N-ω-fluoropropyl-2β-carboxymethoxy-3β-(4-iodophenyl) nortropane (FP-CIT) single-photon emission computed tomography (SPECT) images (**C**) and ^123^I-meta iodobenzylguanidine (MIBG) cardiac scintigraphy (**D**). PSP patients have a significantly lower uptake of FP-CIT in the caudate nucleus and putamen than iNPH patients and normal individuals. In PD and LBD, there is nearly no MIBG uptake in the myocardium, while normal MIBG uptake in the myocardium is shown in PSP. iNPH – idiopathic normal pressure hydrocephalus; PD – Parkinson’s disease; LBD – Lewy body dementia.

*Progressive supranuclear palsy.* PSP is the most difficult condition to differentiate from iNPH (67) because the gait disturbances are difficult to distinguish. In cases of typical PSP exhibiting supranuclear oculomotor disorder, cerebral MRI shows atrophy of the midbrain tegmentum. This is referred to as the hummingbird sign, since the medial rostral section of the atrophied midbrain looks like a hummingbird’s beak in the sagittal plane. Atrophy of this region is accompanied by enlargement of the third cerebral ventricle and cerebral aqueduct. Atrophy is further seen in the pons and cerebellum, particularly in the frontal lobe of the cerebral hemispheres. DAT-SPECT shows abnormalities, while MIBG myocardial scintigraphy findings are largely normal (Figure 4). However, there are reports of PSP cases that lack typical findings, such as midbrain tegmentum atrophy, and instead exhibit DESH findings, making them difficult to distinguish from iNPH using imaging (68). Differential diagnosis using Aβ42 or tau levels is also difficult, and at present, there are no useful methods or biomarkers.

## Treatment prognosis due to comorbid neurodegenerative disease

The pathophysiology of iNPH is still under investigation, and although CSF shunting is expected to improve symptoms, the degree of improvement is variable. The most important factors determining shunting prognosis are shunt-related complications and co-existence of comorbid degenerative diseases. The shunt-related complications: infection, shunt dysfunction, headache due to CSF overdrainage, and subdural effusion and hematoma, which often occur in the early postoperative period, can be reduced by adequate management. Their early treatment can prevent serious sequelae, and recovery can be expected. On the other hand, neurodegenerative comorbidities can affect long-term outcomes. No report accurately indicates the prognosis of neurodegenerative comorbidities with iNPH. A Swedish Hydrocephalus Quality Registry study using a self-assessed modified Rankin Scale or subjective improvement found that vascular comorbidity co-existence did not deteriorate outcomes 2-6 years after shunting (69). However, a Japanese nationwide hospital-based survey evaluating the risk factors associated with shunt placement in patients with modified Rankin Scale grade 2 showed an association with comorbid chronic ischemic lesions (odds ratio [OR], 2.28; 95% confidence interval [CI], 1.11-4.67; *P* = 0.025). In the same study, patients with modified Rankin Scale grade 3 at study entry had an association with comorbid AD (OR, 3.02; 95% CI, 1.44-6.31; *P* = 0.003) (70). iNPH patients with comorbid AD can improve following shunt surgery. However, outcomes (particularly cognitive) may be less satisfactory compared with “typical” iNPH patients (59), particularly in the long term. In another report, the improvements across all applied criteria (including the modified Rankin Scale), were maintained for three years after treatment in a low p-tau group. In the high p-tau group, improvement was observed in the early stage, peaked at six months, and gradually declined later to or below preoperative levels (71).

Gait disturbance is an essential common symptom of iNPH and several neurodegenerative disorders. Disorders that have to be considered for the differential diagnosis of iNPH include PD and atypical parkinsonian disorders. Because of these overlapping features, it is not uncommon for individuals with idiopathic PD or secondary parkinsonism to be suspected of having concomitant iNPH. DAT imaging is believed to be helpful for this purpose, but as a possible explanation, the mechanical effect exerted on the striatum by ventriculomegaly ultimately downregulates dopaminergic transporters, which may improve after shunt (72). Comorbidity with atypical parkinsonian disorders (73,74), MSA, PSP, CBD, or LBD affects the long-term prognosis of shunting in wide-based gait with staggering walk, lateral instability executive dysfunction (59), and apathy within 3 years of the treatment.

## Conclusions

Comorbidities are important in iNPH management because they can explain symptoms, co-exist with iNPH, and affect outcome and prognosis. The workup of patients suspected with iNPH should include evaluation of the history and clinical symptoms, clinical syndrome, and comorbidities. Although there are still no highly sensitive and specific CSF biomarkers that can predict the effect of a CSF shunt intervention, the potential of Aβ42, p-tau, total tau, NfL, and LRG has been reported in multiple studies. However, these biomarkers do not specifically reflect the pathology of iNPH. Even so, total tau and p-tau may differentiate iNPH from AD, and Aβ42 may differentiate iNPH from healthy individuals. Thus, a combination of these biomarkers may improve the diagnostic accuracy of iNPH.
